# Low-cost modular chromatography column rack and vial holders

**DOI:** 10.1016/j.ohx.2022.e00388

**Published:** 2022-12-10

**Authors:** Ruby L. Schaufler, Niall C. Slowey

**Affiliations:** Texas A&M University, United States

**Keywords:** Gravity-driven column chromatography rack, Open-source hardware, Modular and flexible, Purify, Compound separation, Fraction collection

## Abstract

Gravity-driven chromatography columns are used in scientific, engineering, medical, and industrial fields to separate desired compounds from solutions. Running multiple columns simultaneously saves time and improves procedural consistency. Though column chromatography is widely used, to meet their laboratory needs many investigators must resort to designing and fabricating custom racks for holding their chromatography columns. We have created a robust column rack design, with collection vial holders, that is easily made, inexpensive to build, and may be easily adapted to fit experimental needs. The column holder can be made to hold various sizes of columns (and can be interchanged as necessary); the height of columns above collection vials can be precisely set; and the design is modular, so the rack and vial holders can be expanded to accommodate the desired numbers of columns and the numbers and sizes of vials used to collect fractions eluted from each column. Importantly, the rack is made of inexpensive, readily–available materials and the fabrication is straightforward. Here we present details of the rack’s features, a list of materials, and instructions for making it. We hope our design will help investigators who perform gravity-driven column chromatography.

## Graphical abstract

**Figure:**
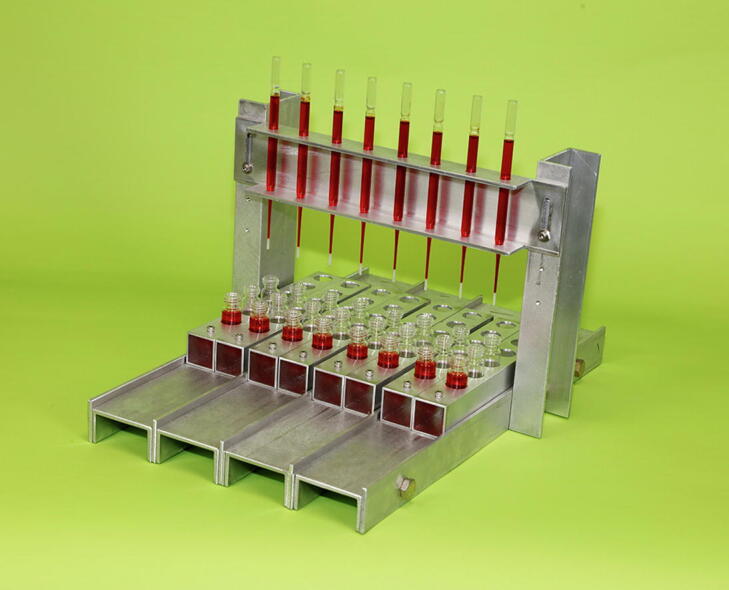

## Specifications table

**Table:** 

Hardware name	Chromatography column rack and vial holders
Subject areas	•Chemistry and Biochemistry
	•Geosciences
	•Environmental, Planetary, and Agricultural Sciences
	• Medical (e.g., Pharmaceutical Science)
	•General
Hardware type	Sample preparation: separation and/or purification
Open-Source License	Creative Commons by Attribution 4.0 International
Cost of Hardware	$334 USD ($59 USD for metal materials; ∼$275 USD for construction depending on services available)
Source File Repository	A PDF format Design File is included as a Supplementary material for this article (this same file is also available on the Open Science Framework website *https://osf.io/wc428/*)

## Hardware in context

The design presented here was built for geochemical analysis of alkenones, an organic molecule found in marine sediments that is used as an indicator of past seawater temperatures [e.g., refs [1], [2], [3], [4]]. To obtain alkenones for analysis, total lipid extracts were separated into three fractions; a-polar, mid-polar (containing alkenones), and polar fractions using our gravity-driven column chromatography rack. Gravity-driven chromatography racks are used in a very wide variety of other applications as well. The following are a few examples: organic geochemistry [5], [6], geobiology [7], paleoceanography [8], [9], paleoclimatology [10], [11], petroleum geology [12], [13], [14], [15], [16], stereochemical enantiomer self-disproportionation [17], [18], [19], trace metal analysis [20], [21], [22], organic and physical chemistry [23], the food and drug industry [24], [25], [26], [27], [28], [29], [30], and medical sciences [31]. When performing column chromatography, multiple samples of solutions composed of complex mixtures often need to be purified and/or separated to analyze desired compound(s). Even with small batches of samples, it is convenient to simultaneously run multiple columns that require the same separation scheme. A rack for holding several samples is necessary because it enables more than one sample to be processed at the same time, which improves the consistency of results and also saves the technician time.

Few chromatography column racks are available for purchase, and those that are available have limitations. A rack may only hold a single column, ring stands or wall mounts may be needed to support the rack, plastic construction materials may be incompatible with reagents or solvents, or there may be limitations in the dimensions of columns that the rack can accommodate. Moreover, available racks usually do not include holders for collection vials so the racks are awkward to use. To overcome these limitations, many laboratories use custom-built column chromatography racks, but designs for custom-built racks are not readily available. Moreover, the steps required to fabricate such racks typically involve complex tools (e.g., milling machine) operated by specially trained operators, so fabrication costs are high.

We have designed a column chromatography rack that has several advantages. It is robust and made to be used on a regular basis in a laboratory setting. Optimal construction materials can be selected to withstand contact with reagents/solvents used during a given laboratory’s column separation process, as well as to take advantage of inexpensive, available construction materials. The modular design can be easily adjusted to accommodate the number of columns and collection vials used. The rack’s column holder can also be easily adapted to hold any given size chromatography column. The same rack can accommodate more than one size of column by simply switching a single piece of the rack (referred to as the column holder below); to do this involves only two screws be loosened – we used cap screws that can be tightened/loosened by hand (no tools required). Thus, one column chromatography rack can handle a range of column sizes and therefore be used for a wide array of chromatography schemes. Our design also includes dedicated vial holders that maintain collection vials in a stable, upright position. The integration of the rack and vial holders makes it easy for the user to align collection vials directly beneath the columns and enables multiple fractions to be readily collected from each column. Collection vials associated with one column can also be moved independently of vials associated with other columns.

## Hardware description

• *Chromatography column rack and vial holders for simultaneous separation of multiple samples, which leads to greater consistency and reduces operator time.*
• *The modular design is easily adapted to match the number of columns routinely used.*
• *The rack and vial holders can be built out of any solid material and also adapted to various column sizes, so the rack is compatible with the reagents/solvents and columns needed for any given laboratory method.*
• *Simple, readily available materials and tools are necessary to construct the hardware, so it is easy and inexpensive to build.*

We present here a flexible design for building a modular chromatography column rack. It is readily scaled so a user can build a rack that holds any desired number and size of columns. We chose to build our rack out of aluminum because it is compatible with the use of dichloromethane in our chromatography method (plastic materials would dissolve if they come into contact with dichloromethane) and aluminum materials are readily available, inexpensive, and easy to work with. However, users can construct the rack from other materials (e.g. wood, plastic, glass, etc.) if they are better suited for use with the reagents employed in the user’s separation scheme.

**Fig. 1:**
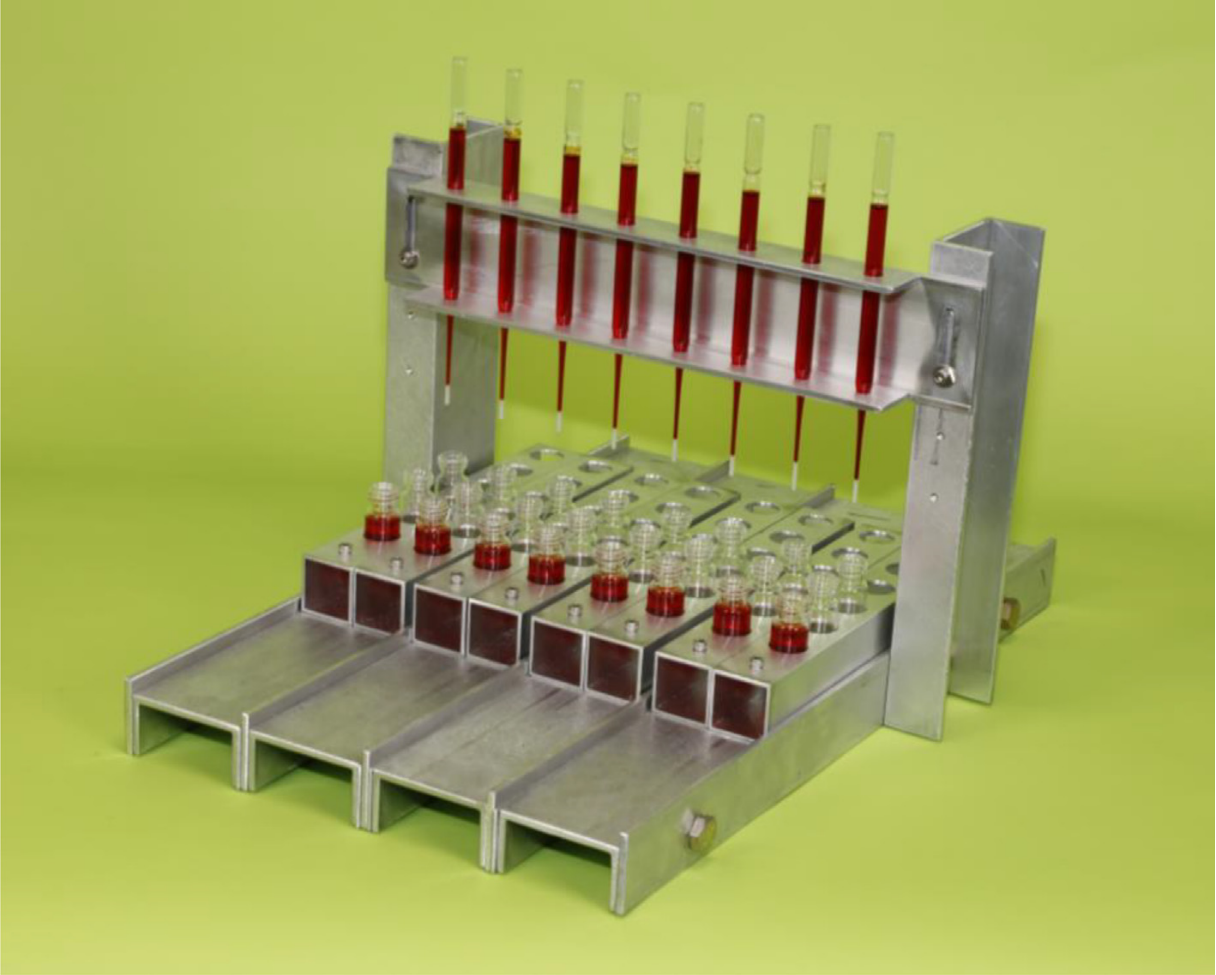
Our modular column chromatography rack that holds eight standard 5¾” long Pasteur pipettes in a stable, upright position as they are used for gravity-driven chromatographic separations and purifications. Moveable vial holders are used to align collection vials beneath each column and hold them in a stable fashion. See the text and the **Design File** for details of dimensions and relative positions of the components use to build the rack.

**Fig. 2:**
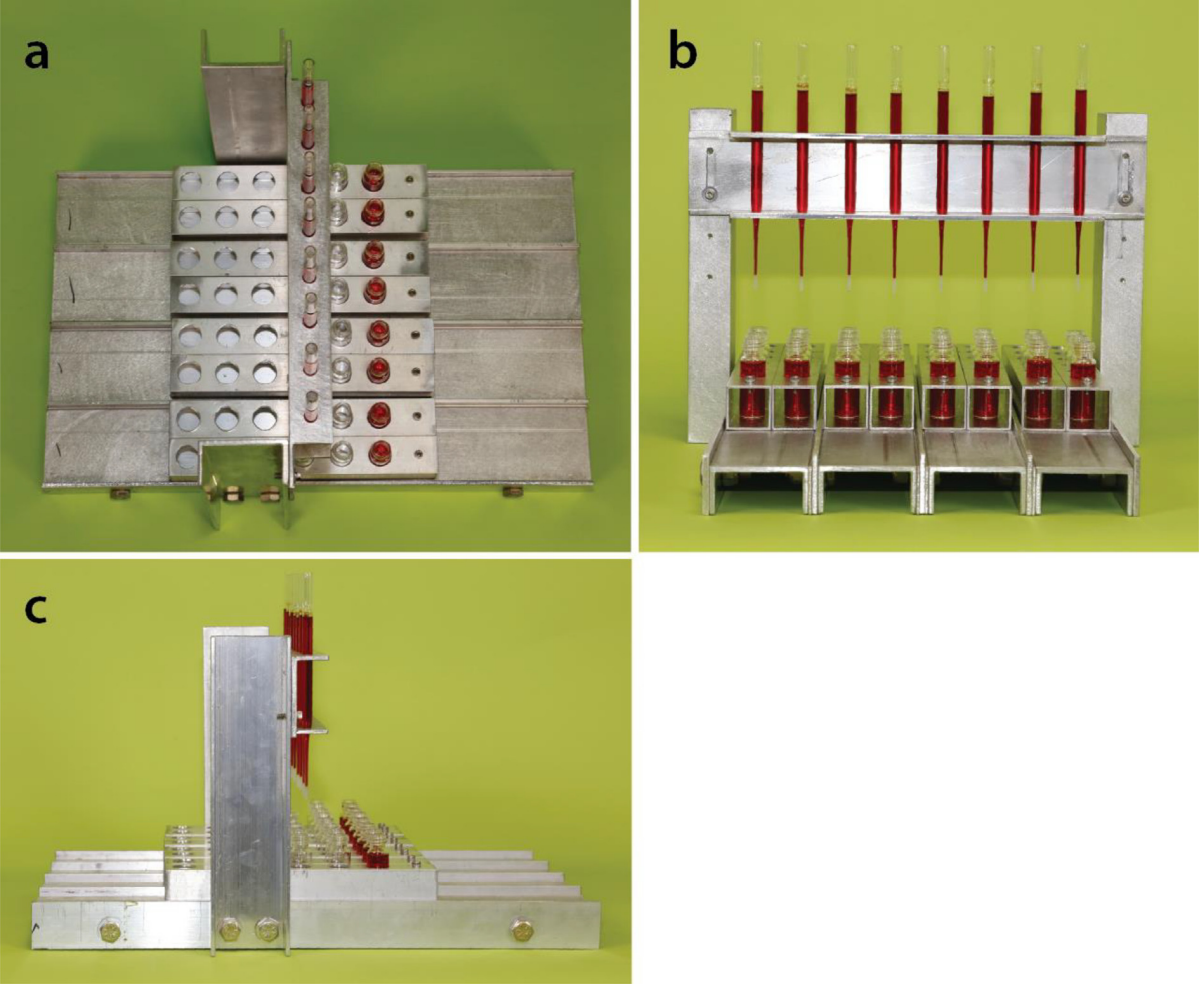
Top **(a)**, front **(b)**, and side **(c)** views of the column chromatography rack. The modular nature of the design is apparent, as well as the guides that help the user align collection vials beneath the columns and enable the user to move an individual holder without interfering with the other holders. See the text and the **Design File** for details of dimensions and relative positions of the components use to build the rack.

As shown in Fig. 1, Fig. 2, and the **Design File**, our rack is strong and lightweight (6.8 lbs/3.1 kg), and has a stable base that does not require any external support. The lightweight and robust nature of the design make it ideal for use not just on a laboratory bench, but in a fume hood, on a ship, or in the field as well. The height of columns above the collection vials can be conveniently adjusted and the U–channel construction makes all portions of the columns visible, which enables the user to better monitor how the purification/separation process is proceeding. Holders keep the collection vials stable; each holder can hold several vials and slide independently along a fixed track in the rack, so it is easy to position collections vials directly beneath each column. This design is for manually operated column separations. However, one can envision automating parts of this system by using a motorized drive or step motor to either add solutions to the columns or to advance to position of the vial holders beneath the columns. Many examples and designs of automated systems are available in open-source journals [32], [33], [34], [35].

## Design files

A PDF format file with the **Design File** for the column rack and vial holders is available as a supplementary HardwareX file for this article, as well as on the Open Science Framework website. It includes a top, bottom and side view of each rack component along with their specific dimensions and relative positions. We built our rack and the vial holders using aluminum parts held together with common bolts and screws (see below). Other types of materials could be used to make the rack compatible with specific laboratory needs or to take advantage of locally available materials.

**Table:** 

Design file name	File type	Open-source license	Locations of the file
*chromatography_column_rack.pdf*	*PDF*	*Creative Commons by Attribution 4.0 International*	1) Supplementary material*s* 2) *Open Science Framework**https://osf.io/wc428/*

## Bill of materials

We used the following list of materials to build an aluminum rack that holds eight standard 5¾″ disposable Pasteur pipettes used as chromatography columns and eight collection vial holders, which can each hold six 4 ml (1 dram) collection vials. Note: while metal parts are listed below, plastic or wooden parts may be substituted if they are easier to obtain or more compatible with reagents and other analytical needs. Note: the units provided here in the manuscript and in the **Design File** are given in imperial units due to the availability of materials and tools in our area. This design is modular so the size of the rack can be easily customized to accommodate the needs of the user. For convenience: 1″ is equal to 2.54 cm.

**Table:** 

*Designators*	*Component*	*#*	*Unit cost (USD)*	*Total cost (USD)*	*Source of materials*	*Material type*
• *Base* • *Column holder* • *Column holder supports*	*Aluminum U-channel* *1″* × *2″* × *96″*	*1*	*$20.50*	*$20.50*	*Grainger* *Item #6ALY4*	*Aluminum*
*Dividers to separate vial holders on base*	*Flat bar stock* *⅛”x1¼”x96″*	*1*	*$9.23*	*$9.23*	*Grainger* *Item #6ALX3*	*Aluminum*
*Spacers in base to allow vial holders to slide*	*Flat bar stock* *_1/16_″x1″x72″*	*1*	*$10.43*	*$10.43*	*Lowe’s* *Item #215707*	*Aluminum*
*Vial holders*	*Square tube* *⅞” inside square (1″ outside) × 72″*	*1*	*$11.54*	*$11.54*	*Grainger* *Item #6ALR6*	*Aluminum*
*Bolts to fasten base together*	*#10*–*32 cap screws (50/Pk)* *#10 dia., 32 TPI thread, ½” length*	*1*	*$3.18*	*$3.18*	*Monsterbolts.com*	*Stainless Steel 18*–*8*
*Nuts to fasten base together*	*#10*–*32 Hex nut (25/Pk)*	*1*	*$1.63*	*$1.63*	*Monsterbolts.com*	*Stainless Steel 18*–*8*
• *Screws to fasten column holder supports to base* • Vial holder handles*	*#6*–*32 Socket head cap screws (10/Pk) (or wing nuts)* *#6 dia., 32 TPI thread, ½” length*	*1*	*$0.78*	*$0.78*	*Monsterbolts.com*	*Stainless steel 18*–*8*
*Washers for screws that fasten column holder to base*	*Flat washers (25/Pk)* *#10 dia.*	*1*	*$0.87*	*$0.87*	*Monsterbolts.com*	*Stainless steel 18*–*8*

* Recommended but not necessary.

## Build instructions

What follows are brief instructions for building an aluminum rack for holding eight chromatography columns and eight collection vial holders. This design is modular so the size of the rack can be easily customized to accommodate the needs of the user. For example, the number of chromatography columns that the rack holds can be changed by adding/reducing the number of U-channel pieces included in the base and by extending/shortening the length of the column holder. The size of the holes used to hold the columns and vials (as well as the dimensions of the U-channel that serves as the holder) can be modified to accommodate any size chromatography column. Our rack was built in a metal working shop on the campus of Teas A&M University, but more readily available tools such as a hand-held saw, electric miter saw, table saw, bandsaw, and hand-held drill (with table vice) or drill press could have been used to make it. Sanding burrs and sharp edges of cut surfaces is highly recommended to produce smoother and safer edges on the rack.

The units provided here in the manuscript and in the **Design File** are given in imperial units due to the availability of materials and tools in our area. One inch is equal to 2.54 cm.

### Base and vertical supports

**Fig. 3:**
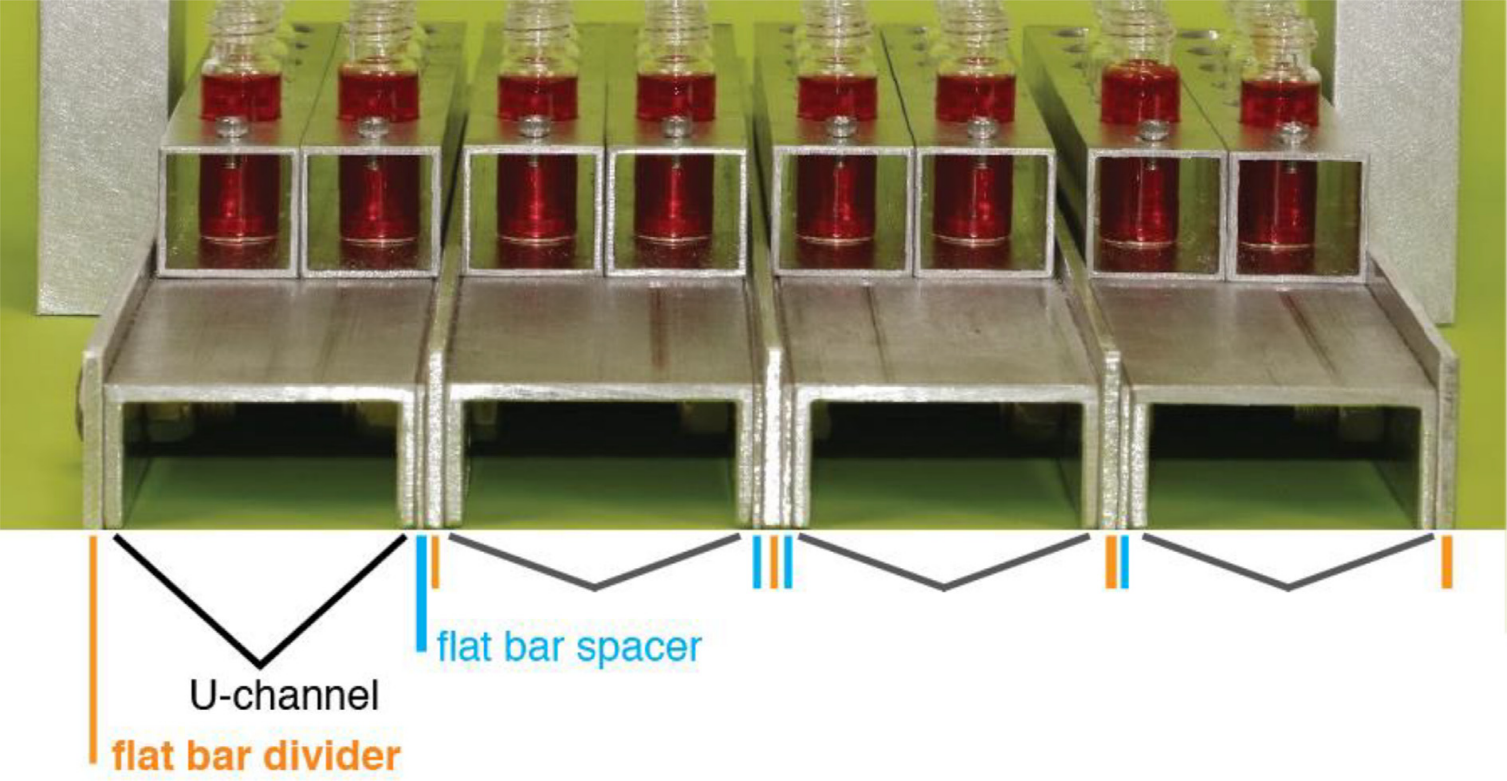
Flat aluminum bar stock and U-channel are used to create the base of the chromatography column rack. Aluminum U-channel is used to support the vial holders and is separated by flat bar dividers that guide the alignment of the collection vials with the chromatography columns. Flat bar spacers are placed alongside each piece of U-channel to allow the vial holders to slide easily along the U-channel track.

**Fig. 4:**
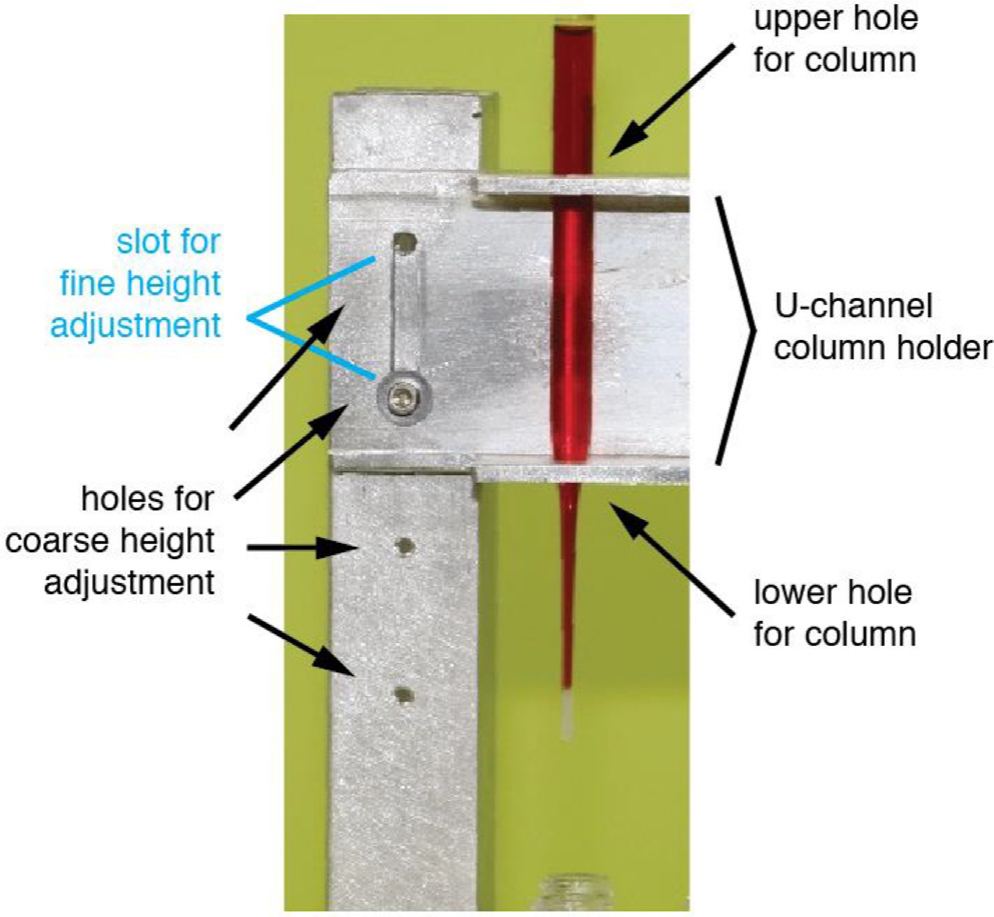
Slots and holes are used to attach the chromatography column holder to the vertical supports of the rack. These holes and slots allow the user to adjust the height of the chromatography columns above the collection vials; to do this involves only loosening two cap screws. The diameter of holes on the top of the column holder must be slightly larger than the diameter of the column so it can pass through the hole and also remain in a stable upright position, while the diameter of holes on the bottom of the column holder must be larger than the tip of the column but smaller than the diameter of the column, so columns rest in the holder above the collection vials.

The chromatography column holding rack has a base and two vertical supports that ensure the column holder is held in a firm and stable upright fashion, and the base presents a flat surface that supports the collection vial holders and it has guides that maintain the vial holders in proper alignment with the columns. Detailed aspects of the base and column holder supports are illustrated in Fig. 1, Fig. 2, Fig. 3, Fig. 4 and the **Design File**.

To build the base’s surface, cut 4 pieces of aluminum U-channel, 4 pieces of flat aluminum (^1^∕_16_″x1″), and 5 pieces of flat aluminum (^1^∕_8_″x1¼”), where every piece is 14½” long. The smaller pieces of flat aluminum will be used as spacers between the U-channel, and the larger pieces of flat aluminum will be used as track dividers that guide the alignment of the collection vials with the chromatography columns.

Mark the length-wise and cross-wise centers of these 13 pieces (do this on the 1″ sides of the U–channel). Then drill two ^13^∕_64_″ diameter holes through both the 1″ sides of the U–channel and the flat aluminum pieces (refer to Fig. 1, Fig. 2**c** and the **Design File**). The centers of the holes in each piece should be ½“ up from their bottom edges and 1¾” from the front and back edges of the rack. Cap screws will be passed through these holes to fasten the components of the base together (described below). Note: any size screw that fits the dimensions of the base can be used to assemble it (adjust diameter of holes and fasteners to fit screws). We have included the measurements we used here as a working example.

Two additional holes are needed on each side of the base to attach the two vertical supports for the chromatography holder (refer to Fig. 2**a and 2c** and the **Design File**). These ^13^∕_64_″ diameter holes pass through the flat piece of aluminum and adjacent outer edge of U-channel that form the two sides of the base. It is important to note that the vertical supports are not centered on the sides of the base, rather, they should be offset ¾” towards the back half of the base. This placement of the vertical supports ensures that when the column holder is attached, each column will be centered length-wise over the base of the rack.

Once all of the holes are drilled through the pieces of the base, line up the pieces as shown in the **Design File** and Fig. 3. Then, pass #10–32 cap screws through the holes drilled in each piece (2 holes per flat aluminum piece and 4 holes per U-channel piece) and fasten them with washers and nuts. Note: the two pieces of flat aluminum and the outer sides of the U-channel pieces that are to be on the sides of the base will have 2 additional holes, through which cap screws will pass to attach the vertical supports for the column holder to the base.

The next step is to make the vertical supports for the chromatography column holder. Cut two 8″-long pieces of aluminum U-channel that will serve as the supports. Then drill two ^13^∕_64_″ diameter holes through the 2″ side on one end of each piece of U–channel. These holes must align with the two ^13^∕_64_″ diameter holes previously drilled through the mid-portion of each side of the base so cap screws can be passed through them to fasten the vertical supports to the base of the rack. Refer to Fig. 2**c** and the **Design File**.

To connect the column holder to the vertical supports you will need to drill ^5^∕_32_″ holes through the front 1″ side of the vertical supports at a height such that when columns are in the holders the bottom of the columns will be at the desired height above the collection vials. We drilled 4 of these holes 1″ from the top of the support and separated them vertically by 1″. Refer to Fig. 2**b**, Fig. 4, and the **Design File**. Once all of the holes are drilled through the vertical supports, line up the ^13^∕_64_″ holes on the vertical supports with the ^13^∕_64_″ holes on the sides of the base as shown in Fig. 2**a,** Fig. 2**c,** and the **Design File** and pass #10–32 cap screws through the holes and fasten them with washers and nuts.

### Column holder

The column holder holds eight Pasteur pipettes used as chromatography columns (refer to Fig. 1, Fig. 2, Fig. 4 and the **Design File**). To build the column holder, cut a 11^7^∕_8_″-long piece of aluminum U-channel. Designate one of the 1″ sides of the U-channel as the top of the holder and the other as the bottom of the holder. To hold the columns in place, 8 pairs of holes must be drilled in the top and bottom 1″ sides of the U–Channel, where the centers of each pair of holes are aligned vertically. The diameter of holes on the top of the column holder must be slightly larger than the diameter of the column so it can pass through the hole and also remain in a stable upright position, while the diameter of holes on the bottom of the column holder must be larger than the tip of the column but smaller than the diameter of the column, so columns rest in the holder above the collection vials. Distances between the centers of each pair of holes are such that the tip of each chromatography column is aligned with the centers of the collection vial beneath it (refer to **Design File**). These spacings can be adjusted to accommodate the dimensions of the materials available to build the rack and the size of the columns used.

We drilled ^9^∕_32_″ diameter holes on the top and ¼” diameter holes on the bottom of the column holder to accommodate standard 5¾” Pasteur pipettes. The sizes of these holes can be adjusted to accommodate the diameter of any column. Potential modification options to accommodate different shaped columns can be found in section 5.4.

As described in **section 5.1**, the column holder is held above the base of the rack by vertical supports and there are a series of ^5^∕_32_″ holes separated by 1″ in the front side of each support to allow the height of the column holder to be adjusted. In addition, we put a centered slot on each side of the column holder to allow for finer height adjustments (refer to Fig. 2**b, 4** and the **Design File**). We used cap screws that can be tightened/loosened by hand (no tools required) to attach the column holder to the vertical supports.

Note: if the dimensions of the columns and collection vials to be used will never change, then only one hole needs to be drilled in each side of the column holder and each vertical support (the placement of these holes must accommodate the dimensions of the columns and vials used).

### Vial holders

**Fig. 5:**
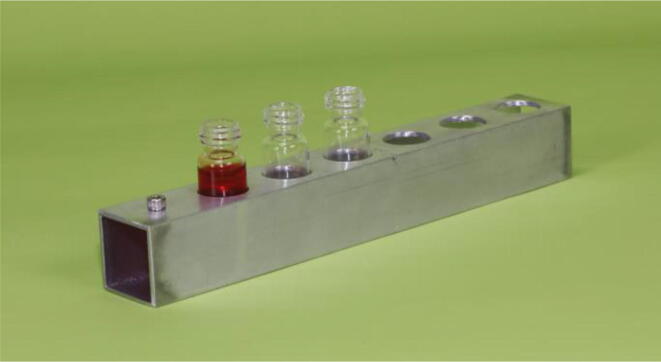
An individual vial holder that can holds up to six 4 ml (1 dram) collection vials beneath each chromatography column. See the text and the **Design File** for details of dimensions and relative positions of the components used to build the rack.

We built one collection vial holder for each chromatography column (Fig. 1, Fig. 2, Fig. 5). We built holders that can hold six 4 ml collection vials. However, the number of vials that each holder accommodates can be adjusted to the user’s needs.

To build a vial holder, cut a 7″ long piece of square aluminum tubing. On the side of the square tube that will serve as the top of the holder, holes will be drilled to attach a handle and to insert each collection vial (refer to Fig. 5 and the **Design File**). Drill a centered hole ½” from one end of the vial holder through which a cap screw can be passed to serve as a handle (we used a #6–32 cap screw so the diameter of the hole was ^5^∕_32_″). Drill vial holes which have their centers at 1″ intervals starting at the center of the handle hole. We drilled ^19^∕_32_″ diameter holes, which is slightly larger than the diameter of our 4 ml collection vials.

### Potential modifications

**Building material:** Rather than using aluminum to build the rack, users can construct it from other materials (e.g. wood, plastic, glass, etc.) if they are more readily available or better suited for use with the reagents employed in the user’s separation scheme.

**Column holder:** The design was made for Pasteur pipettes (or similarly shaped columns).

The column holder can be adapted to accommodate columns with a valve/stopcock (e.g. a burette). Rather than having a hole on the top of the column holder, create a u-shaped space that is open to the front edge of the holder. This u-shaped space would allow the column to be inserted into the rack even though it has a wide, irregularly-shaped valve/stopcock. If desired, small holes on either side of the u-shape can be drilled to insert a wire, piece of metal, or other material to hold the upper portion of the column in place.

If a long column is used that is not adequately supported by the U-channel specified in the plan, the vertical supports on the rack can be made longer and either a wider U-channel can be used, or a second U-channel (or piece of angle stock) can be used to support the column at two widely spaced positions.

## Operation instructions

Gravity-driven column chromatography is used in various settings for different applications. Place chromatography columns with contents required for a given chromatography method into the holder and they will be held in a stable, upright position while they are used for gravity-driven chromatographic separations and purifications. Place the number of collection vials required for a given chromatography method in each vial holder, then slide the vial holder on the surface of the rack’s base until a collection vial is aligned underneath each chromatography column.

## Validation and characterization

We constructed a rack and vial holders using the designs presented here, and regularly use them in our laboratory. They have proven to be both easy to use and effective for simultaneously doing gravity-driven column chromatography separations of multiple samples.

## Declaration of Competing Interest

The authors declare that they have no known competing financial interests or personal relationships that could have appeared to influence the work reported in this paper.
