# Supplementary material for: Low-cost modular chromatography column rack and vial holders
Source: HardwareX. 2022 Dec 10;13:e00388. doi: 10.1016/j.ohx.2022.e00388 (PMC9791163; doi:10.1016/j.ohx.2022.e00388)

# COLUMN HOLDER

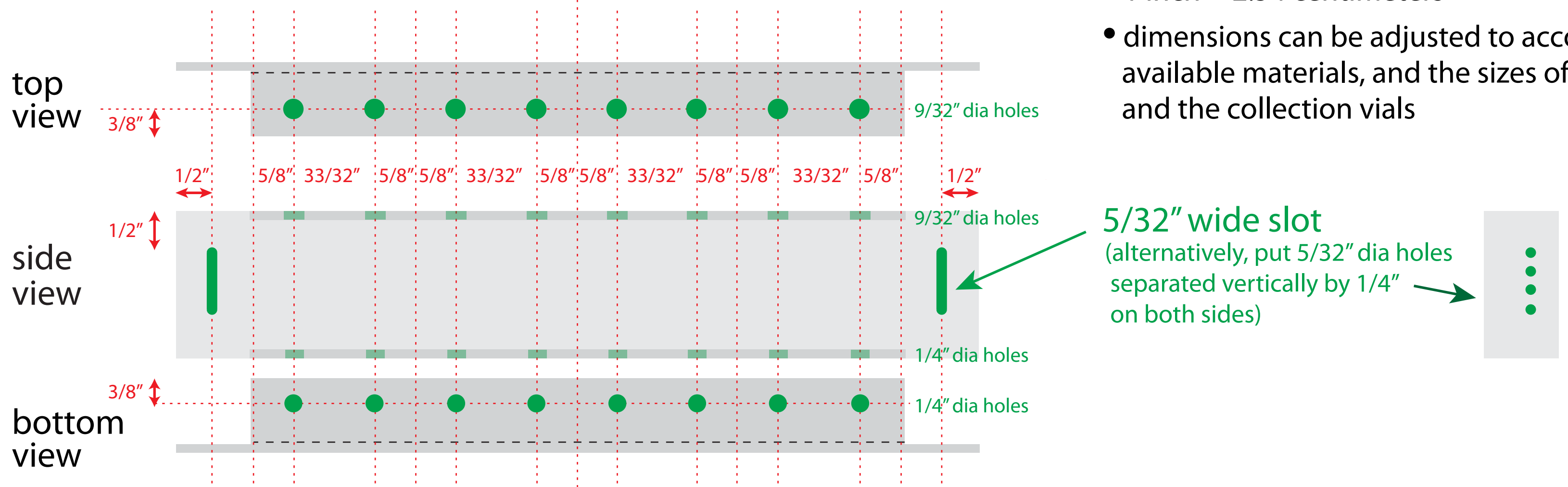


Notes on dimensions:

- 1 inch = 2.54 centimeters
- dimensions can be adjusted to accommodate available materials, and the sizes of the columns and the collection vials

# BASE

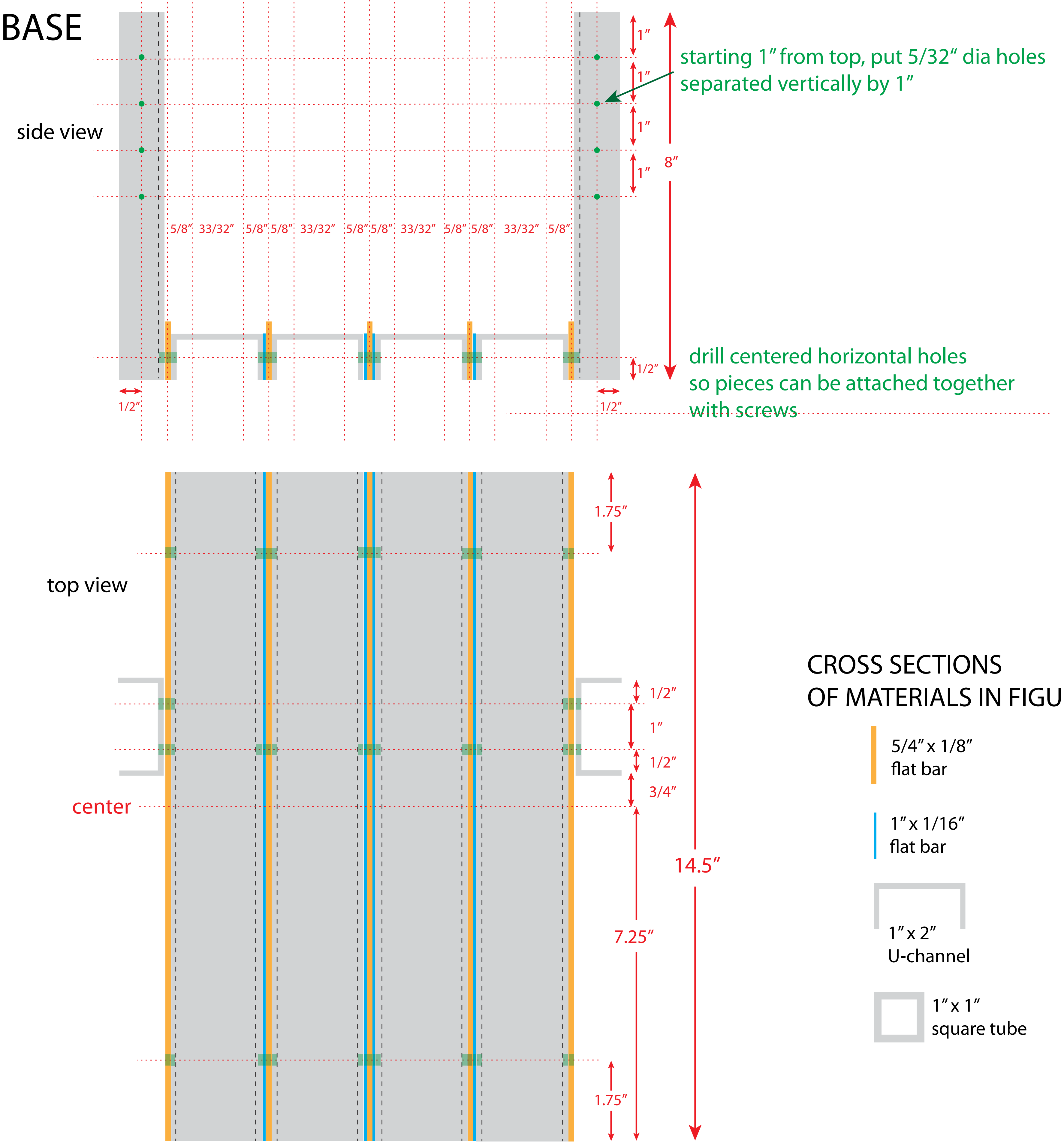


## CROSS SECTIONS OF MATERIALS IN FIGURE

5/4" x 1/8" flat bar

1" x 1/16" flat bar

1" x 2" U-channel

1" x 1" square tube

# VIAL HOLDERS (make 8 of these)

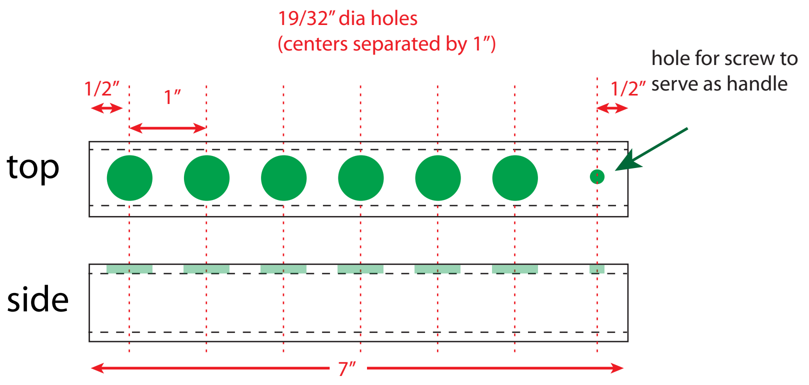

Supplement: Supplementary data 2 [file mmc2.pdf]
